# Lumbopelvic Stabilization with Two Methods of Triangular Osteosynthesis: A Biomechanical Study

**DOI:** 10.3390/jcm13164744

**Published:** 2024-08-13

**Authors:** Niklas Grüneweller, Julia Leunig, Ivan Zderic, Boyko Gueorguiev, Christian Colcuc, Dirk Wähnert, Thomas Vordemvenne

**Affiliations:** 1 Bielefeld University, Medical School and University Medical Center OWL, Protestant Hospital of the Bethel Foundation, Department of Trauma and Orthopedic Surgery, Burgsteig 13, 33617 Bielefeld, Germany; niklas.grueneweller@evkb.de (N.G.); christian.colcuc@evkb.de (C.C.); dirk.waehnert@evkb.de (D.W.); 2 AO Research Institute Davos, Clavadelerstrasse 8, 7270 Davos, Switzerland; ivan.zderic@aofoundation.org (I.Z.); boyko.gueorguiev@aofoundation.org (B.G.)

**Keywords:** dorsal pelvic ring, lumbopelvic instability, biomechanics, triangular fixation

## Abstract

(1) **Background**: Pelvic fractures, and particularly instabilities of the dorsal pelvic ring, are becoming increasingly prevalent, particularly in orthogeriatric patients. Spino-pelvic triangular osteosynthesis is an effective approach to achieve sufficient stabilization in vertically unstable fractures. This study compares two types of osteosynthesis: the conventional one and a novel instrumentation where the iliosacral screw is placed through a fenestrated iliac screw. (2) **Methods**: Sixteen artificial osteoporotic L5+pelvis models with an unstable sacral fracture have been instrumented with either an iliac screw connected with a rod to a L5 pedicle screw and an iliosacral screw (TF) or a fenestrated ilium screw connected with a rod to a L5 pedicle screw and an iliosacral screw passing through the fenestra of the iliac screw (TFS). Biomechanical testing was performed using cyclic loading until failure. (3) **Results**: Both configurations yielded comparable results with regard to initial stiffness, implant loosening, and cycles to failure. The TFS exhibited markedly higher values for cycles to failure and markedly lower values for loosening. However, due to the characteristics of the artificial bone model, these findings were not significant. (4) **Conclusions**: The novel triangular fixation systems demonstrated comparable results to the standard triangular osteosynthesis configuration.

## 1. Introduction

The posterior pelvic ring may exhibit instability for a number of reasons, including traumatic instability following high-energy trauma, instability caused by fragility fractures, and tumor-induced instability. Over the past few decades, there has been a significant shift in the demographic profile of patients with pelvic fractures [1,2,3]. One of the primary causes of pelvic instability in developed countries are fragility fractures [2,3,4,5]. Consequently, pelvic fractures are a prevalent manifestation of osteoporosis. As a result, women are disproportionately affected by pelvic fractures [1]. Looking specifically at this high-risk population, a FRISBEE (Fracture Distribution in Postmenopausal Women) study found an incidence of pelvic fractures of 117 per 100,000 women per year in the 60–69 age group, 244 in the 70–79 age group, and 787 in the 80–89 age group [6]. Other risk factors for pelvic insufficiency fractures, besides female sex and osteoporosis, include obesity, hypertension, diabetes, renal insufficiency, vitamin D deficiency, nicotine abuse, and hypocalcemia [7].

Moreover, this type of fracture has undergone a significant transformation, evolving from simple (such as coccygeal fractures, stable fractures of the ilium bone, and fractures of the upper and or lower pubic branch) to more complex varieties. Between 1991 and 2013, the AO/OTA (AO Foundation/Orthopaedic Trauma Association) type B fractures exhibited a five-fold increase, while type C fractures exhibited a doubling in this period [2].

High-grade instabilities (multiple planes), such as U- and H-shaped fragility fractures, AO/OTA type C fractures, and spino-pelvic dissociations, necessitate surgical stabilization. Particularly in the orthogeriatric population, certain risk factors such as comorbidities and reduced physical and mental fitness exist that complicate the treatment of pelvic fractures [7,8]. Another important issue is the reduced implant anchorage due to osteoporotic bone quality, which increases the risk of implant loosening and further fracture collapse [7,8].

Consequently, two main concerns of minimally invasive or percutaneous surgical procedures are the reduction in complications (e.g., blood loss, hematoma, wound infection, soft tissue damage, and postoperative pain) and biomechanical stability to improve healing success. Minimally invasive or percutaneous surgical procedures ensure a sufficient level of pain relief and the possibility of early mobilization under full weight-bearing in the orthogeriatric population [9]. Consequently, the surgical treatment of dorsal pelvic ring instability has undergone a significant evolution in the recent decades, shifting from a conservative approach to an operative intervention [10,11]. Operative stabilization allows for early mobilization, which is beneficial for patient recovery. Studies have demonstrated that surgical stabilization of pelvic ring injuries, compared to non-operative treatment, facilitates early mobilization, reduces early mortality, and improves long-term outcomes in polytraumatized patients [12]. In addition, surgical techniques have evolved from open to minimally invasive and percutaneous procedures, which is especially beneficial for the other pediatric population. The combination of minimal soft tissue damage (with reduced intraoperative blood loss, reduced postoperative pain, reduced risk of infection) and biomechanical stability (reduced risk of implant failure, full weight-bearing) is therefore essential [5,13]. Additionally, new implants and techniques have been introduced to optimize the treatment of these conditions. A number of techniques exist for the stabilization of the posterior pelvic ring, including percutaneous iliosacral screw fixation [14], plate fixation [15,16], transiliac or transsacral bar fixation [17,18], and spino-pelvic fixation [19,20]. Several innovative approaches attempt to further optimize treatment, such as transsacral S1-S2 screw fixation with cerclage augmentation [21] or the use of pedicle screw rod systems to stabilize the dorsal and anterior pelvic ring [22,23]. In instances of high-grade instability or significantly reduced implant anchorage due to osteoporosis, triangular spino-pelvic fixation can be recommended [13,19,24,25]. Efforts have already been made to make this procedure less invasive [13,26].

The aim of this study was to compare the biomechanical performance of two spino-pelvic fixation methods using an established artificial pelvis model. Both fixations utilized an iliosacral screw and an ilium screw connected via a rod to a L5 pedicle screw (Silony Medical AG, Frauenfeld, Switzerland). The novel aspect of this study is the use of a uniquely designed fenestrated iliac screw (Silony Medical AG, Frauenfeld, Switzerland) which allows for the placement of the iliosacral screw through this fenestra, thereby providing an angular stable construct for the dorsal pelvic ring. By using a screw-head adapter for the ilium screw, the construct is expanded to a spino-pelvic fixation. This construct is biomechanically compared to the conventional construct where the iliosacral screw is placed separately next to the ilium screw.

## 2. Materials and Methods

### 2.1. Implants

**Figure 1:**
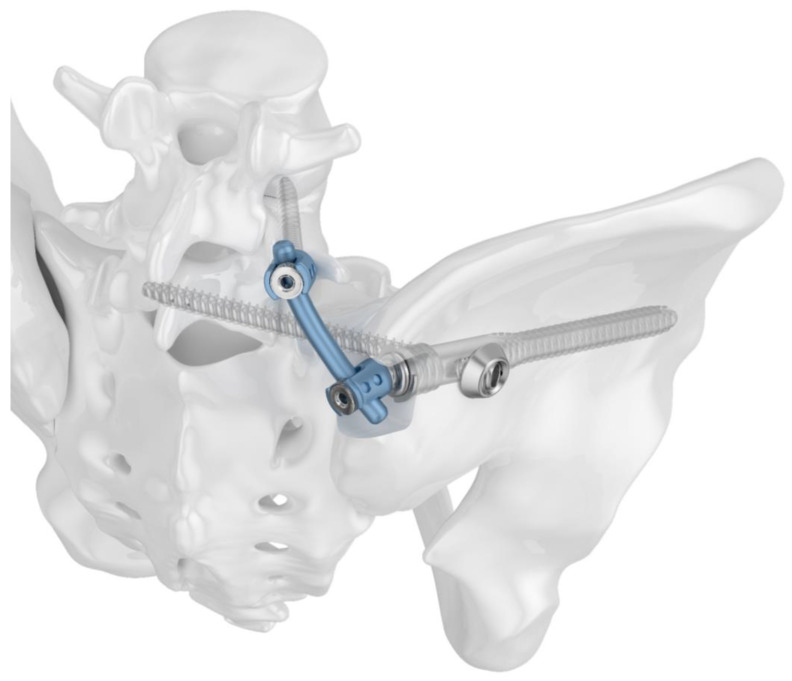
Image of the triangular fixation system (TFS) configuration in Group I: a fenestrated iliac screw and an iliosacral screw with a premounted washer stabilize the dorsal pelvic ring. Lumbo-pelvic stabilization is achieved by connecting the polyaxial head adapter of the iliac screw to a pedicle screw rod system.

**Figure 2:**
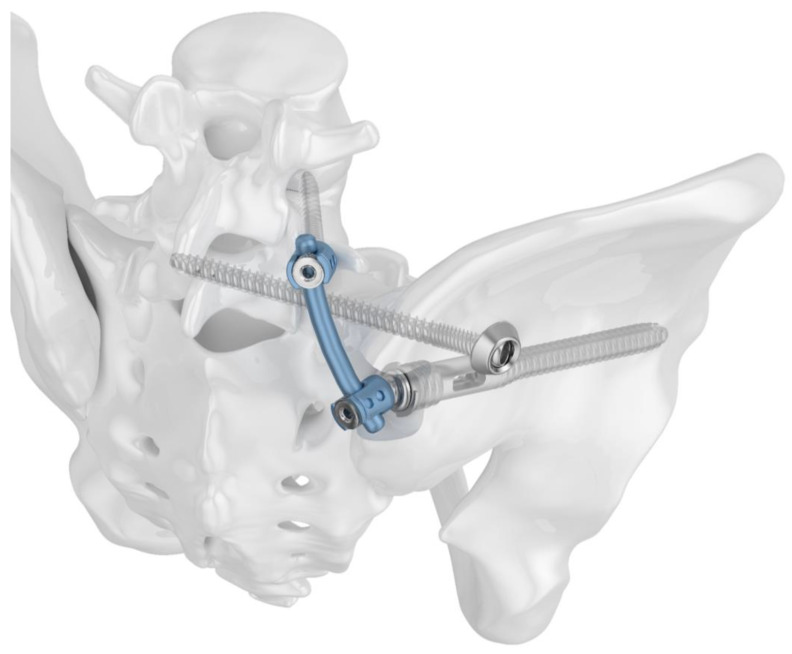
Image visualizing the configuration of Group II (TF): in contrast to Group I, the iliosacral screw is placed separately from the iliac screw in a more cranial position.

Two different implant configurations for a triangular lumbo-pelvic stabilization were tested. All of the used implants were manufactured from titanium alloy Ti6Al4V. Group I (TFS) was stabilized with the triangular fixation system TriFix (Silony Medical AG, Frauenfeld, Switzerland). This was composed of a fenestrated iliac screw with an anterior screw portion with 9.2 mm diameter and a fenestrated portion with 14 mm diameter, and an iliosacral screw with a preloaded washer. An aiming arm device was used to insert the iliosacral screw through the fenestra of the iliac screw. A polyethylene inlay in the fenestra provided quasi-angle stable fixation. The connection between the pelvic ring and the 5th lumbar vertebral body was made using the polyaxial head adapter on the iliac screw and a standard pedicle screw rod system (Figure 1). Group II (TF) was stabilized with the same implants; however, the iliosacral screw was placed separately from the iliac screw in a more cranial position (Figure 2).

### 2.2. Bone Model

This study involves the use of 16 (8 per group) artificial pelvic bones (LS4060, Synbone AG, Zizers, Switzerland) that mimick osteoporosis. This bone model has been employed in numerous biomechanical investigations of the posterior pelvic ring, yielding valuable insights [27,28].

### 2.3. Fracture Model and Instrumentation

An osteotomy in zone 1, as defined by the Denis classification, was performed on the right side of each sacrum using a band saw and a customized cutting guide with the objective of achieving consistent vertical paraforaminal fracture lines. The symphysis and left sacroiliac joint were widely cut to disrupt the pelvic ring [27]. The specimens were randomly assigned to either the triangular fixation system (Group I—TFS) or to conventional triangular fixation (Group II—TF).

In both groups, the sacroiliac joint was fixed with wood screws to emulate ossification and fusion, a common occurrence in orthogeriatric patients, and to focus forces on the sacral fracture [27]. All of the fractures were anatomically reduced prior to fixation. Custom-made drill guides were used to ensure standardized instrumentation and comparable screw positioning in each specimen. Instrumentation was performed with the manufacturer’s appropriate instruments and according to the manufacturer’s instructions.

**Figure 3:**
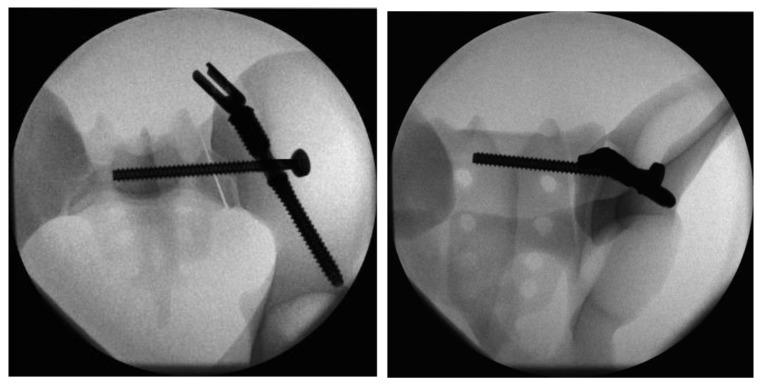
Radiographs in two planes of the triangular fixation system with the sacroiliac screw positioned through the fenestra of the iliac screw.

In Group I, iliac screw insertion was the first step during instrumentation of the triangular fixation system. For this purpose, a 3.2 mm guide wire was inserted into the ilium over the custom-made drill guide starting from the posterior iliac spina under radiographic guidance. Once the correct wire placement was confirmed, the screw hole was prepared by drilling and tapping. The iliac screw was then inserted to the correct depth over the guide wire. For the next step of the iliosacral stabilization, the system’s aiming arm was attached to the iliac screw. This allows for the iliac screw to interlock with the sacroiliac screw. The guide wire for the sacroiliac screw was placed with the help of the attached aiming arm under radiographic guidance. Once the optimal position had been attained, the wire was drilled over and the sacroiliac screw (length 100 mm) was inserted. After the removal of the aiming arm, the polyaxial head was connected to the iliac screw to allow for lumbopelvic stabilization (Figure 3).

**Figure 4:**
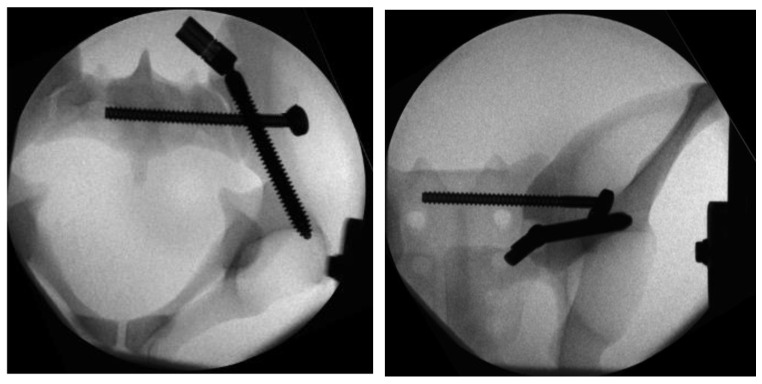
Radiographs in two planes of the conventional triangular fixation with the sacroiliac screw separate from the iliac screw in a more cranial position.

In Group II, the instrumentation was similar to Group I. First, the iliac screw was placed as described. The main difference was in the placement of the sacroiliac screw that was placed separately without the use of the aiming device of Group 1. The guiding wire for the iliosacral screw was placed under radiographic control using a custom-made drill guide. The iliosacral screw was placed next to the iliac screw in a more cranial position to the iliac screw (Figure 4).

In both groups, lumbopelvic stabilization was achieved by connecting the iliac screw via a polyaxial head adapter to a pedicle screw in the 5th lumbar vertebra with a standard rod. The 5th lumbar vertebral body and the sacrum were rigidly attached to the machine actuator (see below). A polyurethane foam was placed between the 5th lumbar vertebra and the sacrum as a spacer and replacement for the intervertebral disk.

### 2.4. Biomechanical Testing

**Figure 5:**
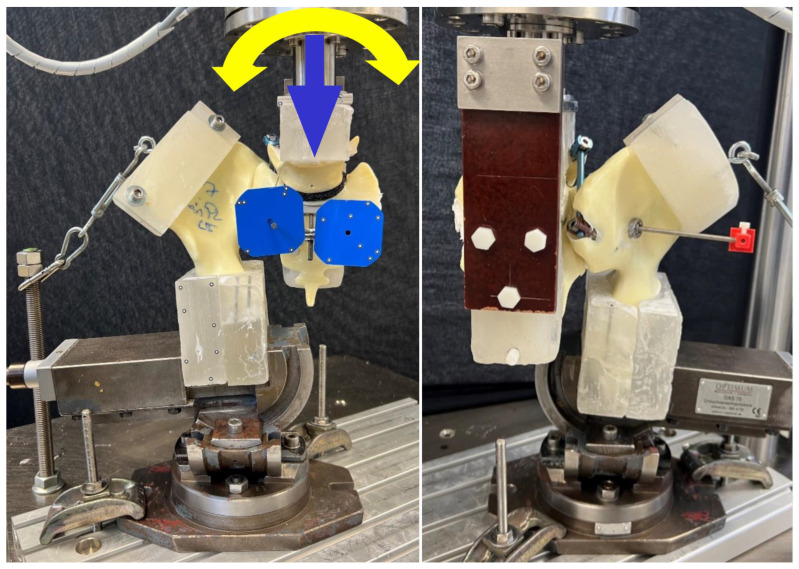
Setup with a specimen mounted for biomechanical testing. View from anterior (**left**) and lateral (**right**). Blue and yellow arrows indicate axial and torsional load applications, respectively.

Biomechanical testing followed a previously published protocol [27]. The biaxial servo-hydraulic material testing machine MTS 858 MiniBionix (MTS Systems, Eden Prairie, MN, USA) was used. It was equipped with a 5 kN/50 Nm load cell. The specimens were positioned in an upright standing orientation. The distal portion was fixed to the base of the machine using a clamp and an X–Y table (Figure 5). The latter allowed for the mediolateral and anteroposterior sliding of the specimen while mounted. Each specimen was clamped during testing. An L-shaped frame fixed to the posterior part of the sacrum by screws through the neuroforamina was used to attach the proximal part of the specimens (5th vertebral body and sacrum) to the machine actuator with the load cell. A clamp connecting the machine frame to the iliac crest was used to simulate muscle tension. A polymethylmetacrylate (PMMA) block was fixed to the iliac portion of the specimen as an anchoring device for the clamp. Optical markers were positioned on the sacrum medially and laterally on the fracture line and on the iliosacral screw for motion tracking.

Prior to the biomechanical tests, the specimens were preloaded with 15 N of muscle tension. The loading regime began with an axial compression ramp starting with 15 N to a maximum of 100 N with a rate of 8.5 N/s. Following this, synchronically cyclic axial and torsional sinusoidal loading at a frequency of 2 Hz was performed until failure. The torsional torque was coupled to the axial load by a constant factor of 1/200. Therefore, the maximal axial loading was increased by 0.05 N per cycle starting from the initial maximal load of 100 N. Similarly, torsional loading was increased by 0.00025 Nm per cycle starting from 0.5 Nm external rotation. The test was stopped when the actuator displaced 30 mm from the starting position.

### 2.5. Data Evaluation

The data from the test machine (axial displacement, axial load, angle of twist, and torque) were acquired by the machine controllers at a rate of 128 Hz. Initial stiffness was determined from the increasing slope of the load–displacement curve of the quasi-static test ramp in the load range 30–60 N. Motion tracking was performed using two 12 MP cameras (Aramis SRX, GOM GmbH, maximum accuracy 0.004 mm) that continuously recorded the marker positions at 50 Hz. The motion of the iliosacral screw tip perpendicular to its axis within the sacrum was calculated as the sacral screw cutout from the motion tracking data. In addition, the tilt of this screw in the iliac bone was evaluated as screw loosening in the iliac bone. Both parameters were assessed at four intervals after 2000, 4000, 6000, and 8000 cycles. To accommodate for specimen settling, comparisons were made with the corresponding values from the third test cycle. Construct failure was defined via the number of cycles to failure when a combination of 2 mm sacral screw tip cutout and 2° of screw loosening in the iliac bone was achieved. Maximum axial compressive loads were used for all evaluations. Radiographs were taken in anteroposterior projection using a controlled C-arm (Siemens ARCADIS Varic VC10A, Siemens Healthineers, Erlangen, Germany). For reference, the first image was taken at the beginning of the cyclic test, followed by images every 500 cycles. The radiographs were used to characterize the location and mechanism of failure.

### 2.6. Statistics

SPSS software (V. 23, IBM SPSS, Armonk, NY, USA) was used for statistical analysis of the parameters of interest. Mean value and standard deviation (SD) were calculated for each of the parameters of interest. For initial stiffness and cycles to failure, statistical differences between the groups were detected using Independent-Samples *t*-tests, and for longitudinal data, these differences were assessed with General Linear Model (GLM) Repeated Measures (RM) tests. Both statistical tests were employed based on given normal data distribution as proved by the Shapiro–Wilk test conducted beforehand. Both tests, Independent-Samples and RM, presume a non-paired study design, which is given by the artificial specimens used. A significance level of 0.05 was employed for all of the statistical tests.

## 3. Results

### 3.1. Initial Stiffness

The mean initial stiffness was 61.4 N/mm (SD 17.2 N/mm) for the triangular fixation system (Group I—TFS) and 60.9 N/mm (SD 12.2 N/mm) for the conventional triangular fixation (Group II—TF). This difference was not significant (*p* > 0.999).

### 3.2. Sacral Screw Cutout and Screw Loosening in Iliac Bone

**Figure 6:**
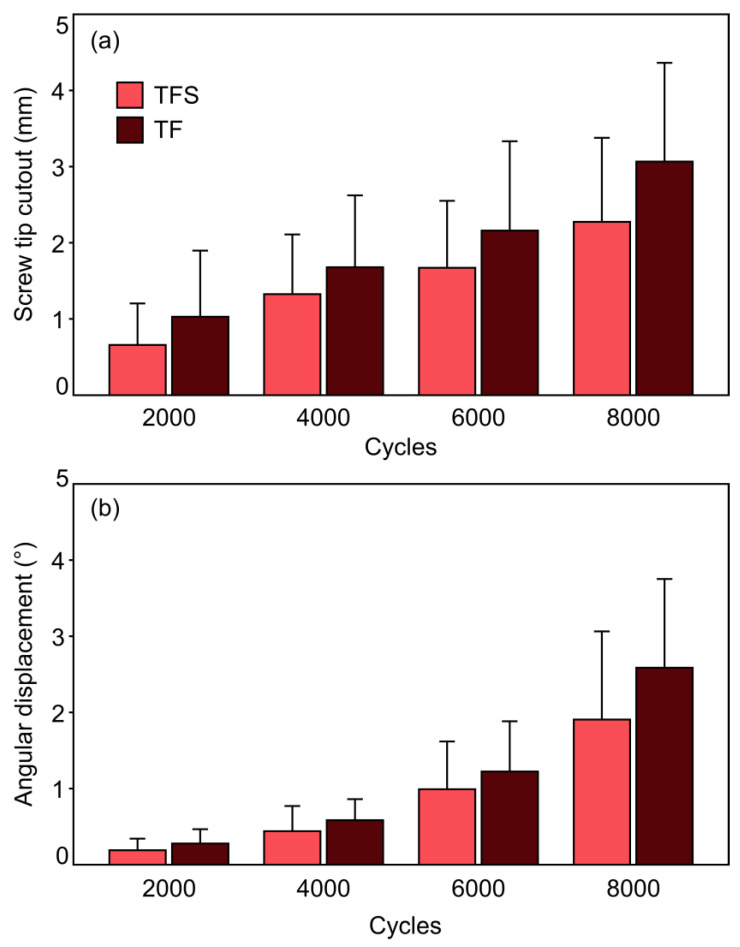
Sacral screw cutout (**a**) and screw loosening in iliac bone (**b**) presented after 2000, 4000, 6000, and 8000 cycles for both groups in terms of mean value and standard deviation.

Figure 6 presents the values for the parameters of the sacral screw cutout and screw loosening in the iliac bone evaluated over the first 8000 cycles at four intermittent time points. In both groups, both the sacral screw cutout and screw loosening in the iliac bone demonstrated a significant increase over the number of cycles (*p* ≤ 0.005). The TFS group showed a 3.5-fold increase in mean sacral screw cutout and the TF group showed a 3.0-fold increase between cycles 2000 and 8000. In regard to the mean screw loosening in iliac bone, the TFS group demonstrated a 10.1-fold increase and the TF group demonstrated a 9.3-fold increase between cycles 2000 and 8000. There were no significant differences between the two groups for either of these parameters (screw cutout *p* = 0.334; screw loosening in the iliac bone *p* = 0.344), although the TFS group had lower values for both the sacral screw cutout and screw loosening in the iliac bone. The difference between the TFS and TF groups for mean sacral screw cutout was +56.4% at 2000 cycles, +26.7% at 4000 cycles, +29.3% at 6000 cycles, and +34.8% at 8000 cycles, whereas this difference for mean screw loosening in the iliac bone was +46.9% at 2000 cycles, +33.0% at 4000 cycles, +23.4% at 6000 cycles, and +35.7% at 8000 cycles.

### 3.3. Cycles to Failure

**Figure 7:**
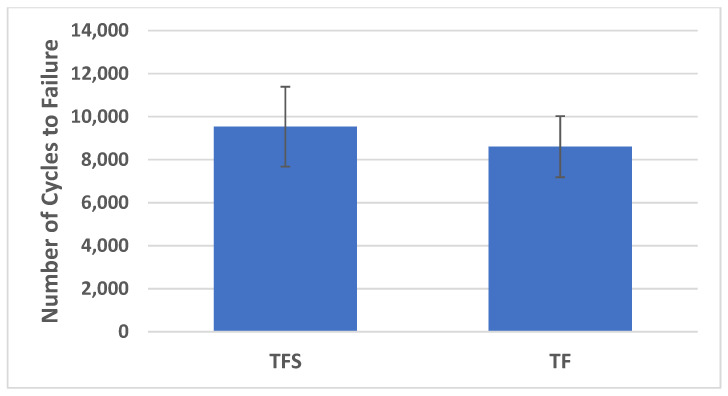
Cycles to failure for both groups (TFS, TF) presented as mean value and standard deviation.

The mean number of cycles to failure was 9534 (SD 2006) for the TFS group and 8605 (SD 1536) for the TF group (Figure 7). This difference was not significantly different (*p* = 0.350). The TFS group demonstrated 10.8% higher mean cycles to failure compared to the TF group.

### 3.4. Catastrophic Construct Failure Mode

**Figure 8:**
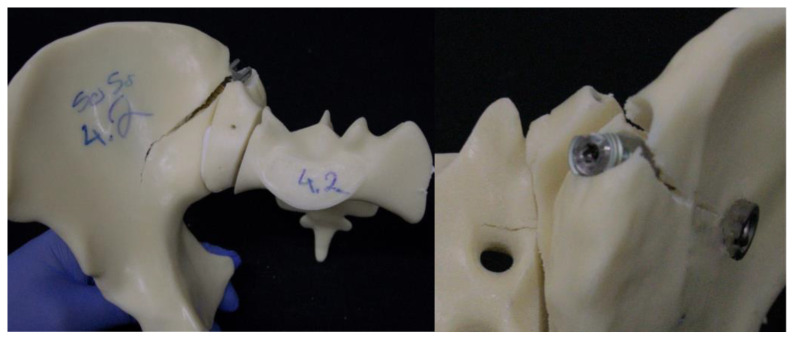
Catastrophic failure pattern in the TFS group, particularly the failure in the trajectory of the iliac screw.

**Figure 9:**
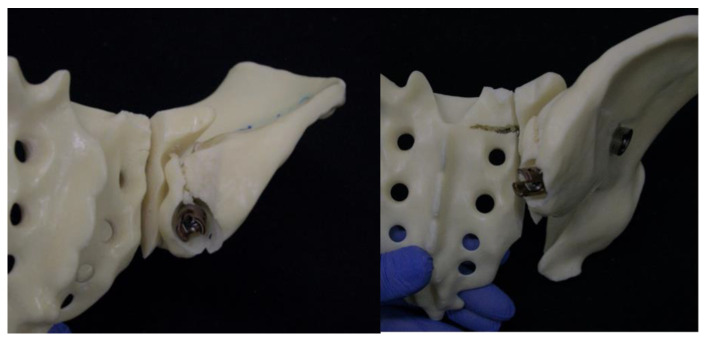
Catastrophic failure pattern in the TF group, particularly the failure in the trajectory of the iliac screw and the sacroiliac screw.

Figure 8 and Figure 9 visualize the catastrophic construct failure mode in both groups. The specimens in both groups failed around the implants in addition to failure at the fracture plane. In particular, the hard and brittle outer structure of the artificial bone model resulted in failure in the area of the implant entry points and trajectories.

## 4. Discussion

Demographic shift has resulted in an elevated prevalence of fragility fractures of the pelvic ring, encompassing complex and unstable fractures of the dorsal pelvic ring. Overall, there is no consensus on the surgical management of these instabilities, with some of the literature suggesting the use of spino-pelvic stabilization to achieve maximum stability for early mobilization.

The current study compares the biomechanical characteristics of conventional triangular fixation versus a newly designed percutaneous and modular triangular fixation system for the dorsal pelvic ring. The results of this work indicate comparable biomechanical findings regarding the initial construct’s stiffness, implant loosening, and cycles to failure. However, the catastrophic failure mode was not comparable with the clinical findings. This discrepancy and the non-significant marginally higher values for the cycles to failure and lower values for implant loosening in the triangular fixation system group may be attributed to the mechanical characteristics of the artificial bone model.

Triangular osteosynthesis, as described by Josten et al., has become a popular treatment option for unstable fractures of the dorsal pelvic ring [29]. This configuration of implants provides both vertical stability—by stabilizing the axis between the lumbar spine (L4, L5) and the crista iliaca—and rotational stability—by placing an iliosacral screw or plate. The initial 34 cases treated with this technique were presented by Schildhauer et al. [19]. Among them, 28 were polytraumatized, with an average age of 35 years. Patients were permitted to bear weight immediately postoperatively. Seventeen patients began with immediate progressive weight-bearing after the operation and reached full weight bearing after 23 days on average. Only one of these patients exhibited implant loosening [19]. Although triangular osteosynthesis of the posterior pelvic ring is a major surgical procedure and also a demanding procedure, the authors conclude that it is a viable option for the treatment of vertically unstable sacral fractures, allowing for early progressive weight-bearing with an acceptable complication rate [19].

Biomechanically, Schildhauer et al. demonstrated the efficacy of their triangular osteosynthesis of the dorsal pelvic ring in a cadaveric single-leg stance model [30]. They compared the triangular osteosynthesis with iliosacral screw osteosynthesis and found that the triangular osteosynthesis exhibited significantly smaller displacement under initial peak loads [30]. Furthermore, all constructs with triangular osteosynthesis demonstrated minimal motion at the fracture site after 10,000 cycles of loading [30]. Consequently, the triangular osteosynthesis demonstrated superior initial stability and dynamic stability [30].

In order to further optimize the technique of the triangular osteosynthesis procedure, additional modifications were made to perform this operation in a minimally invasive or percutaneous manner [5,31]. This was necessary due to the relevant soft tissue complications that are commonly associated with the open procedures [5,32]. In their analysis of 140 cases of pelvic fragility fractures, Rommens et al. found that surgical complications occurred in 19% of the percutaneous treatment group and in 44% of the open treatment group (*p* = 0.006) [8]. Significant differences were observed in the rates of implant failure and surgical revisions. These were more prevalent in the open treatment group compared to the percutaneous treatment group. The rates of implant failure were 0% and 7% (*p* = 0.02), respectively, while the rates of revision surgery were 13% and 29% (*p* = 0.02), respectively [8]. It is of particular importance to pay close attention to the posterior crest of the ilium, particularly in older and thinner patients and in cases of existing soft tissue damage. This is due to the prominence of the iliac screw in triangular osteosynthesis, which can cause complications [12,19].

A number of novel implant designs were developed along with running biomechanical and finite element work to enhance the stability of the dorsal pelvic ring. In general, surgical skills are also of great importance in improving implant fixation. Especially when tightening screws in bone with reduced mechanical capacity, advanced skills are required to achieve sufficient screw purchase without destroying the trabecular bone. The TriFix system increases the stability of screw fixation by providing additional anchoring of the iliosacral screw in the fenestrated iliac screw [33].

Many studies have been conducted with the objective of optimizing iliosacral stabilization. One approach to enhancing the stability of the golden standard—the iliosacral screw—is to perform a cement augmentation procedure in osteoporotic bone. This procedure has been reported to significantly increase stability in several biomechanical studies [34,35,36]. The number of screws placed is also a significant factor in achieving stability. Two iliosacral screws have been shown to provide superior biomechanical results compared to a single screw [37,38]. Nevertheless, the variations in the S1 and/or S2 anatomical corridors frequently preclude the secure screw placement [39,40]. Another crucial factor is the length of the iliosacral implants. Longer screws have been demonstrated to achieve higher biomechanical stability [41,42,43]. The transiliac-transsacral approach—in which screws are placed from one ilium through the sacrum using the S1 or S2 corridor into the contralateral ilium—has been found to provide the highest biomechanical stability [43,44,45]. Considering the aforementioned factors, the iliosacral bar osteosynthesis was introduced and demonstrated favorable clinical outcomes [46,47].

Additionally, efforts were made to optimize the spino-pelvic portion of the triangular osteosynthesis. In their finite element model, Ma et al. found that the fixation of the L4 and L5 segments had no significant effect on the increase in vertical stability [48]. Therefore, fixation of the L5 segment alone is biomechanically non-inferior and can reduce the complications of multi-segmental lumbar fixation [48]. Consequently, the authors conclude that it should be attempted to use the L5 segment for lumbar fixation only [48]. Moreover, Peng et al. demonstrated, in a finite element analysis study, that bilateral triangular fixation exhibited superior stability compared to lumbopelvic fixation and transsacral-transiliac screw fixation for H- and U-shaped sacral fractures [49].

In a recent publication, Sawada et al. presented an interesting approach for the stabilization of a fragility fracture of the pelvis using the E.Spine Tanit (Euros, La Ciotat, France) [50]. This technique combines an iliosacral screw with a sacral anchoring system, allowing for a connection to a rod system. In their case, the authors connected the bilaterally placed anchoring devices to ilium screws; an extension to a L5 pedicle screw would also be possible for a triangular osteosynthesis [50]. An aiming device is used to allow for the placement of the iliosacral screw through the sacral anchoring system. From our perspective, this technique is comparable to the fenestrated iliac screw with the iliosacral screw; however, it requires significantly more material and is associated with a considerably longer operating time (3 h 28 min) [50].

The triangular fixation system (TriFix, Silony Medical AG, Frauenfeld, Switzerland) presented in this study represents an attempt to increase construct stability and address certain clinical needs. These include the percutaneous approach, soft tissue protection, increased patient security, and reduced X-ray exposure. As demonstrated, the combination of the fenestrated ilium screw with an iliosacral screw exhibited superior mechanical stability compared to an iliosacral screw (with an additional short angular stable iliac screw) in the stabilization of unstable sacrum fractures [27]. The modularity of this system allows for the straightforward extension of a spino-pelvic stabilization procedure by simply placing a screw head adapter onto the fenestrated ilium screw. This provides an efficient percutaneous connection to an L5 pedicle screw with an appropriate rod. Furthermore, the fenestrated ilium screw and the screw head adapter are positioned below the posterior iliac crest, offering additional soft tissue protection [51]. To enhance patient safety and minimize radiation exposure, the iliosacral screw is positioned using an aiming device attached to the fenestrated ilium screw. This approach facilitates screw placement, reduces misplacement, and allows for more precise X-ray control. The triangular fixation system, when used in conjunction with this technique, has the potential to enhance stability, reduce soft-tissue complications, and shorten operating times. However, these benefits must be validated through further clinical investigations.

This study is not without limitations. An artificial pelvis model does not accurately reflect normal physiological behavior. The mechanical behavior in particular is very different. An important point is the anisotropy of human bone, which is not found in the artificial model. This affects the entire bone–implant interface. Another major concern is the viscoelastic properties of human bone, which cannot be simulated in artificial models. For example, the bone model used is of brittle nature (especially in the cortical bone) and the response of the model to implantation and testing differs from human bone. In particular, the iliac insertion had to be modified to allow for insertion without destroying the artificial bone model. In addition, these differences result in a catastrophic failure that differs from clinical findings. Another point that differed from the clinical application was the fixation of the iliosacral joint with wooden screws. This procedure allowed for standardization in all specimens, and also somehow represented the situation in the orthogeriatric patient population, where ossifications are quite common. Despite all of these limitations, artificial bone models allow for a standardized comparison of implants and osteosynthesis and several biomechanical studies were conducted using these bone models, allowing for a comparison of the results between studies [27,52]. Therefore, we decided to use this osteoporotic artificial pelvis model. Other crucial elements are the biomechanical testing and the assessment of initial construct stability. However, the application of a cyclic loading regime is more informative with respect to the analysis of biomechanical failure than the use of static testing protocols. Since biomechanical testing cannot simulate healing, it can only determine the initial construct’s stability. The earliest stage of the overall loss of fixation stability is the loosening of the critical parts of the osteosynthesis. Therefore, we decided to evaluate screw loosening/cut-out at in the ilio-sacral region. The experimental setup employed is analogous to those utilized in numerous previous studies in this field, thus facilitating a direct comparison of the outcomes [27,52].

## 5. Conclusions

This study presents the first biomechanical data on spino-pelvic stabilization with a novel triangular fixation system. Overall, from a biomechanical perspective, the new system demonstrates comparable results to the standard triangular osteosynthesis configuration. However, marginal superiority was observed in the loosening parameters and the number of cycles to failure. However, due to the mechanical characteristics (brittle nature) of the artificial bone model, these findings are not statistically significant. Furthermore, the novel triangular fixation systems offer a distinctive degree of modularity and an aiming device for the iliosacral screw, which represents a significant clinical advantage.

## Data Availability

All data are available on request from the corresponding author.
